# The Interpretation of Dyspnea in the Patient with Asthma

**DOI:** 10.1155/2015/869673

**Published:** 2015-12-24

**Authors:** Marc H. Lavietes

**Affiliations:** New Jersey Medical School, 100 Bergen Street, No. I354, Rutgers, Newark, NJ 07103, USA

## Abstract

Physicians have noted dyspnea in severely ill asthmatic patients to be associated with fright or panic; in more stable patients dyspnea may reflect characteristics including lung function, personality and behavioral traits. This study evaluates the symptom of dyspnea in 32 asthmatic patients twice: first when acutely ill and again after an initial response to therapy. Spirometry was performed, dyspnea quantified (Borg scale), and panic assessed with a specialized measure of acute panic (the acute panic inventory (API)) in the 32 patients before and again after treatment. After treatment, questionnaires to evaluate somatization and panic disorder were also administered. When acutely ill, both the API and all spirometric measures (PEFR; FEV1; IC) correlated with dyspnea. Multiple linear regression showed that measures of the API, the peak expiratory flow rate, and female sex taken together accounted for 41% of dyspnea in acute asthma. After treatment, the API again predicted dyspnea while spirometric data did not. Those subjects who described themselves as having chronic panic disorder reported high grades of dyspnea after treatment also. We conclude that interpretations of the self-report of asthma differ between acutely ill and stable asthmatic patients.

## 1. Introduction

Dyspnea is a cardinal symptom of asthma. The notion that fear or panic may contribute to the sensation of dyspnea accompanying acute bronchospasm has been recognized by clinicians and is discussed in the psychiatric literature [1]. Similarly, physicians caring for outpatient asthmatics have long recognized that both personality traits and psychological state as well as pulmonary dysfunction may influence the self-report of dyspnea in stable patients [2–4]. This study examines the following hypothesis: the genesis of dyspnea experienced by acutely ill asthmatic patients and that of stable asthmatic patients may differ.

## 2. Materials and Methods

We studied 32 English-speaking acutely ill asthmatic patients twice: once immediately upon their arrival in the emergency room and again after stabilization. Subjects older than 50 years or younger than 18 were excluded as were pregnant women and those with coexisting chronic heart or lung disease.

Spirometry was first performed upon arrival at the emergency service before administration of any therapy. We used a pneumotachograph affixed to a portable computer (Respitech, Lancaster, Pa). The procedure was repeated (as many as six times) until reproducible expiratory flow and inspiratory capacity measures were obtained. Acceptable forced vital capacity measurements could not be obtained from many subjects with severe airway obstruction and are thus not reported. Dyspnea was assessed with the modified Borg scale [5]. Patients were specifically asked to rank “how much discomfort do you feel with your breathing” on the zero through ten scale. Finally the 17-item acute panic inventory (API), a concise validated screening tool for panic, was administered [6]. Item number five of this inventory focuses specifically upon dyspnea and was thus omitted from the inventory as used in this study [6, 7].

When stable (as promptly as within 2 hours of presentation in some cases but many days later in others) patients reviewed and signed a consent form. Both the protocol in its entirety and the form were approved by our Institutional Review Board. This form encompassed both the initial and follow-up studies. In addition to repeating the three tests performed in the emergency room, subjects completed two additional questionnaires: one: the Barsky somatosensory amplification scale (SSAS), a test of somatization [8]; two: the Spitzer binary assessment (SPTZ) (Y/N) for a clinical diagnosis of panic disorder [9].

We computed from the spirometry tracing peak expiratory flow rate (PEFR), forced expiratory volume in one second (FEV1), and inspiratory capacity (IC). Data with normal distributions are presented as means and standard deviations. Data with skewed distributions are presented as three values: medians and both upper and lower extremes. Bivariate regression analysis was performed with a nonparametric method. Multiple linear regression was then used to evaluate predictors of the Borg scale jointly. We used stepwise, forward, backward, and Mallows' Cp selection methods to construct a parsimonious model. All methods yielded the same model. Since age and BMI were considered potential confounders and had not been selected via the model selection procedure, they were added to the final model. Categorical variables were compared with a one-way analysis of variance. Finally data obtained upon entrance to the study were compared with data obtained at follow-up by paired *t*-test. The SAS 9.3 statistical package was used. All testing was two-sided at the 0.05 significance level.

## 3. Results and Discussion

We studied 18 M and 14 F subjects. Their mean (SD) age was 38 ± 7; their weight (BMI) was 30 ± 9 kg/m^2^. Their median initial Borg score was 5.0 (0.5; 8.0). Initial lung function data showing severe airway obstruction appear in Table 1. Lung functions and their correlations with dyspnea during acute illness appear in Table 1 also. Measures of all lung functions as well as panic (API) correlated closely with acute dyspnea. Note that only 6 of the 32 subjects responded “yes” regarding a positive clinical history of panic attacks. Borg scale scores for “yes” and “no” responders were similar. The best fit multiple linear regression model appears in Table 2. Spearman's *r* for this model was 0.64. Measures of PEFR and API taken together with the categorical variable sex accounted for 41% of the variability within the Borg scale response. While sex was not predictive of dyspnea in the bivariate analysis, it was in the multivariable analysis with women having higher Borg scores than men.

The median Borg score after treatment was 1 (0, 7). The differences between pre- and postvalues for the API and all lung functions (Tables 1 and 3) were significant at the *p* < 0.001 level. Correlations of dyspnea measured after treatment with independent variables appear in Table 3. Lung function and dyspnea did not correlate. Both the API and the binary assessment for the clinical diagnosis of panic correlated with dyspnea in the stable subjects. By contrast, correlation between dyspnea and either the SSAS or the BMI did not reach statistical significance. Only the API however correlated with dyspnea when all variables were entered into the multiple linear regression model.

Dyspnea in acute asthma reflects both uncoupling of inspiratory effort from inspiratory flow (airway narrowing) and hyperinflation. Both uncoupling and hyperinflation are promptly relieved by treatment. Subsequently in stable subjects, lung function is no longer a major determinant of dyspnea.

Anxiety, as measured by the API, is a major determinant of dyspnea in the acutely ill subject. As such it is likely to be a component of acute panic. The API is a simple, brief screening tool designed to evaluate anxiety in severely ill, unstable subjects. To this end, some of our patients when acutely ill resembled panic disorder patients. Many reported difficulty working, speaking, and concentrating. Some described sweatiness and shaking. Two reported extreme symptoms such as the need to urinate or defecate. It is likely that an occasional patient presents with both severe bronchoconstriction and panic, too agitated to participate in a study. We did not encounter such a patient. The fact that only 6 subjects responded “yes” regarding a positive clinical history of panic and that this “yes” response was not linked to high Borg scores in the emergency setting suggests that panic disorder per se is not common in these patients and plays no role in their dyspnea during acute illness.

The API as a measure of anxiety remained a strong predictor of dyspnea after treatment. In addition, panic disorder as assessed by the SPTZ questionnaire correlated with dyspnea when dyspnea was measured after treatment. The observation that the SPTZ correlates with dyspnea in the treated but not the acutely ill asthmatic patient is of interest. The SPTZ is not a definitive diagnostic tool. It is a self-assessment screening tool that identifies patients who believe they qualify for the diagnosis of panic. In the context of this study, the SPTZ identifies patients who see themselves as being emotionally labile and, as such, would be likely to exaggerate unpleasant sensations such as dyspnea. Taken together, these data support two notions: one: the panic experienced in acute asthma does not necessarily reflect a generalized panic disorder; two: the mechanisms producing dyspnea in acute versus stable asthma differ.

Note that preexisting psychological conditions may predispose to asthma or perpetuate the condition. This study does not address preexisting psychological conditions but supports the notion that only panic and airway obstruction account for the dyspnea reported during an acute, untreated asthma attack. By contrast, other psychological factors, not examined here, may be more important in determining the degree of dyspnea in the stabilized asthmatic patient [10–12]. Lastly sex differences in exertional dyspnea, noted previously, are explained by differences in respiratory reserve between men and women [13].

### 3.1. Limitations of This Study

This study population is limited to inner city patients. The absence of suburban and rural subjects or subjects with varied socioeconomic and ethnic backgrounds is a major limitation to a generalized interpretation of the results. Both cultural differences in the perception of symptoms and varied accessibility to health care between persons of differing socioeconomic backgrounds are well known [14, 15]. Lastly a measure of airway inflammation, known to modify dyspnea perception in asthma but not obtained here, could have enhanced the prediction of dyspnea in our subjects [16].

## 4. Conclusion

We conclude that the self-report of dyspnea and spirometry obtained simultaneously provide complementary information for the routine assessment of stable asthmatic patients.

## Figures and Tables

**Table 1:** Bivariate analysis using Spearman correlation to measure association of dyspnea (dependent variable) with lung function and psychometric data obtained in the emergency department (independent variables) obtained simultaneously.

Independent variable	Mean (+SD)	Spearman *r*	*p*
FEV1, % predicted	41 + 22	−0.47	0.006
IC, % predicted	57 + 27	−0.37	0.036
PEFR, % predicted	38 + 23	−0.45	0.009
API	9 (1, 32)	0.55	0.001
SPTZ		0.16	0.370

API = acute panic inventory; FEV1 = forced expiratory volume, one second; PEFR = peak expiratory flow rate; IC = inspiratory capacity; SPTZ = Spitzer binary assessment, administered after treatment only. Group data for API scores are expressed as median (maximum, minimum). For all other variables, group data are expressed as mean + SD.

**Table 2:** Multiple linear regression model to predict correlation with the Borg scale score.

Characteristic	Parameter estimate	95% CI	*p*
Intercept	5.45	(2.24, 8.67)	
Sex (M/F)	1.88	(0.59, 3.17)	0.01
Age	−0.03	(−0.09, 0.02)	0.24
BMI (kg/m^2^)	0.01	(−0.05, 0.07)	0.75
PEFR, % predicted	−0.05	(−0.08, −0.03)	<0.001
API	0.11	(0.06, 0.16)	<0.001

For definition of abbreviations, see Table 1.

**Table 3:** Bivariate analysis, data obtained after treatment, when stable.

Independent variable	Mean (+SD)	Spearman *r*	*p*
FEV1, % predicted	65 + 24	0.11	0.561
IC, % predicted	87 + 22	0.04	0.821
PEFR, % predicted	58 + 25	−0.01	0.979
API	2 (0, 21)	0.59	0.004
SPTZ	6 Y/26 N	0.53	0.002

For definition of abbreviations, see Table 1.
